# A Comprehensive Framework for Evaluating Cycling Infrastructure: Fusing Subjective Perceptions with Objective Data

**DOI:** 10.3390/s25041168

**Published:** 2025-02-14

**Authors:** Kefei Tian, Yifan Zheng, Zhongyu Sun, Zishun Yin, Kai Zhu, Chenglong Liu

**Affiliations:** The Key Laboratory of Road and Traffic Engineering, Ministry of Education, Tongji University, Shanghai 201804, China; 2251090@tongji.edu.cn (K.T.); 2253783@tongji.edu.cn (Y.Z.); 2251128@tongji.edu.cn (Z.S.); vcccyzs@tongji.edu.cn (Z.Y.); 2251051@tongji.edu.cn (K.Z.)

**Keywords:** cycling friendliness evaluation, multi-data fusion, vibration signal processing, computer vision, reveal preference survey

## Abstract

As cities increasingly prioritize green and low-carbon transportation, the development of effective cycling infrastructure has become essential for alleviating traffic congestion and reducing environmental impacts. However, the service quality of bike lanes remains inadequate. To address this gap, this study proposes a multi-data-fusion framework for evaluating bike lane “cycling friendliness”, integrating subjective perceptions with objective metrics. The framework combines survey-based subjective data with digital measurements to enable rapid, large-scale assessments that align with user expectations. Tailored evaluation models are developed based on revealed preference (RP) survey analysis to account for variations among target user groups. Key factors such as road roughness, motor vehicle encroachment, cycling-friendly amenities, and roadside scenery are quantitatively assessed using vibration analysis and computer vision techniques. Validation results reveal a strong correlation between model predictions and subjective evaluations, demonstrating the framework’s reliability and effectiveness. This approach offers a scalable, data-driven tool for optimizing bike route selection and guiding infrastructure upgrades, thus advancing urban cycling transportation.

## 1. Introduction

With the rapid development of urbanization and motorization, traffic congestion and environmental challenges have become increasingly severe worldwide. In 2022, global greenhouse gas emissions peaked at a record 57.4 billion tons of carbon dioxide equivalent. Given that motor vehicles are significant contributors to carbon emissions, identifying and promoting low-carbon transportation options is essential. Slow-moving transportation modes, such as walking and cycling, are gaining popularity globally as sustainable and environmentally friendly travel options. Notably, the global bicycle count reached 1 billion in 2022, underscoring cycling’s significant potential as a green transportation mode. Despite the rapid growth in bicycle numbers, the service level of bike lanes has significantly lagged, resulting in a suboptimal cycling experience. Consequently, the quality of bike lanes has become a critical factor in supporting this green mode of transportation, with their construction and maintenance playing a vital role in promoting cycling culture and reducing carbon emissions.

To evaluate cycling accessibility, the concept of ‘cycling friendliness’ has been introduced as a complement to ‘walkability’ [1]. Recently, terms such as ‘bikeability’, ‘cyclability’, and ‘cycling index’ (used interchangeably) have been applied to assess a community’s cycling-related environment [2]. Researchers have found that providing safe, convenient, and comfortable cycling infrastructure can increase residents’ willingness to cycle, reduce motorized trips, and consequently minimize pedestrian–vehicle conflicts, thus promoting the green, low-carbon development of urban transportation [3]. Existing bike lanes exhibit numerous inadequacies that require improvement. Currently, no reliable and scientific methods exist to assess the strengths and weaknesses of these facilities accurately. Given this situation, a more rigorous and effective approach is needed to scientifically evaluate and analyze the ‘cycling friendliness’ of slow-traffic infrastructure. This approach facilitates the identification of areas for further optimization and refinement in cycling infrastructure based on current conditions, enabling targeted improvements in construction and management.

Numerous recent studies have developed indicator systems to quantify cycling friendliness. Earlier research often relied on direct evaluations, with some researchers gathering road assessment data through surveys and applying data processing methods and reliability tests [4]. Kalvelage et al. used facilitated voluntary geographic information to collect scoring data [5], while others obtained scores through expert measurements [6]. In recent years, research in this field has increasingly introduced more objective and definitive methods to evaluate road cycling friendliness. Some researchers have applied auditing methods to evaluate cycling friendliness: Krenn et al. analyzed the impact of infrastructural compatibility factors on walking and cycling friendliness [7], and Heesch et al. considered factors such as traffic safety, surrounding attractiveness, land use, and related infrastructure [8]. Manton et al. combined mental mapping, stating preference surveys and transport infrastructure inventories to identify influential factors [9]. Other researchers have applied results-oriented methods, directly evaluating each path’s friendliness using accurate data on user choices and inferring the influences of these results on friendliness [10]. Some studies use questionnaires to gather subjective data [11,12], applying traditional methods for questionnaire design, data collection, and analysis [13]. While these various methods consider different influencing factors, they often overlook variations across different types of road functions. Furthermore, these factors differ by geographic population and cannot be uniformly standardized based on previously researched models.

Additionally, various methods have been employed to assess factor scores through data acquisition and analysis. Clifton et al. evaluated road walkability and identified influential factors using consistency percentage as a reliability measure, proposing the Pedestrian Environmental Data Scanning (PEDS) methodology to assess walkability [14]. Cain et al. used Maps-World to assess roads in five countries, focusing on the impact of roadside land use patterns on friendliness [15]. Arellana et al. proposed the Urban Bikeability Index (BI) to assess and prioritize bicycle infrastructure investments, identifying seven key factors through a factor and component selection process [16]. Recent studies have also recognized air quality as an important factor influencing road usability [17].

Recent research on road-friendliness has increasingly leveraged emerging technologies and data sources, such as remotely sensed imagery, manual virtual audits using street view imagery (SVI), and crowdsourcing, to conduct large-scale comparative assessments of bicycle suitability. Zhao et al. audited the friendliness of the urban cycling environment using SVI to assess it [18]. Ye et al. employed street-level image features reflecting cyclists’ spatial perceptions, integrating them with road features at accident sites to assess the cycling environment [19]. Zhang et al. applied spatial design network analysis and a greening quality rating scale to evaluate streetscape accessibility and esthetics [20]. Gong et al. utilized image recognition to assess the streetscape [21]. SVI techniques can automate indicator extraction, though the range of indicators remains limited [22]. The field of autonomous driving is also focusing on real-time semantic segmentation, with several state-of-the-art methods proposed and evaluated [23]. Siam et al. presented a real-time semantic segmentation benchmarking framework with a decoupled design to increase recognition speed [24]. Holz et al. proposed a multi-modal semantic segmentation method, significantly enhancing the accuracy and efficiency of recognition [25]. Regarding data detection, the use of crowdsourced data has become a significant trend in development. Yang et al. enhanced pavement condition monitoring systems by combining limited labeled vibration data with large crowdsourced datasets [26]. Liu et al. proposed a semi-supervised learning (SSL) model to address issues related to incomplete data and multi-vehicle data fusion, which developed a mathematical formulation of the ‘International Roughness Index’ (IRI) using in-vehicle vibration to evaluate driving comfort [27,28].

Although many numerical results appear promising, these studies face significant limitations due to inherent modeling assumptions. Current methodologies rely heavily on subjective evaluations, leading to inaccuracies, time-intensive assessments, and limited scalability. Furthermore, most existing studies fail to account for the variability in functional friendliness evaluations across different types of roads. Conversely, objective evaluation methods often focus on a single element affecting friendliness, resulting in assessments that lack comprehensiveness.

This paper proposes an innovative, fully automated framework for comprehensive road evaluation by integrating subjective model and objective assessment methods. Unlike existing approaches that focus solely on either subjective feedback or objective measurements, this framework synergizes both aspects. The subjective component addresses the actual and differentiated needs of road users, while the objective component enables efficient and rapid cycling road assessments. The results identify current road deficiencies, prioritize improvements, and score factors to guide the development of more satisfactory bike lanes. Additionally, the results serve as a guide for path selection, facilitating road upgrades, and promoting cycling trips.

The rest of this paper is organized as follows: Section 2 introduces the overall framework and specific algorithms of the proposed method, containing both subjective evaluation (RP survey) and objective evaluation (riding roughness, separation method, occupation, scenery, etc.); Section 3 verifies the proposed model using a real case study and illustrates how this method assist the authorities to improve the cycling lanes’ quality; and finally, Section 4 concludes the insight and findings of this paper.

## 2. Methodology

### 2.1. Overall Framework

The cycling friendliness of a road refers to the level of user satisfaction during cycling and represents the quality of service provided by the bike lane. As a critical metric, this measure primarily captures user perceptions. To comprehensively evaluate bike lanes, this study proposes a subjective evaluation framework and evaluates key indicators objectively through advanced data processing techniques, including machine learning. The pre-survey demonstrated that user-perceived elements of friendliness vary significantly across different road types. For example, users prioritize road conditions on roads designed for long-distance travel, whereas roadside scenery is favored on recreational roads. Based on literature research and pre-survey findings, this study categorizes roads into three functional types: access roads, living roads, and recreational roads. To develop the subjective evaluation model, a resident survey was administered to collect user attitudes regarding various road types. Drawing on the literature and pre-survey data, this study identifies key factors influencing cycling friendliness, such as facility support, roadside environment, and road dimensions, which were incorporated into a user questionnaire for evaluation. Following the determination of weights via modeling, signal analysis, and computer vision techniques were employed to extract data and translate detection results into evaluation scores efficiently.

**Figure 1:**
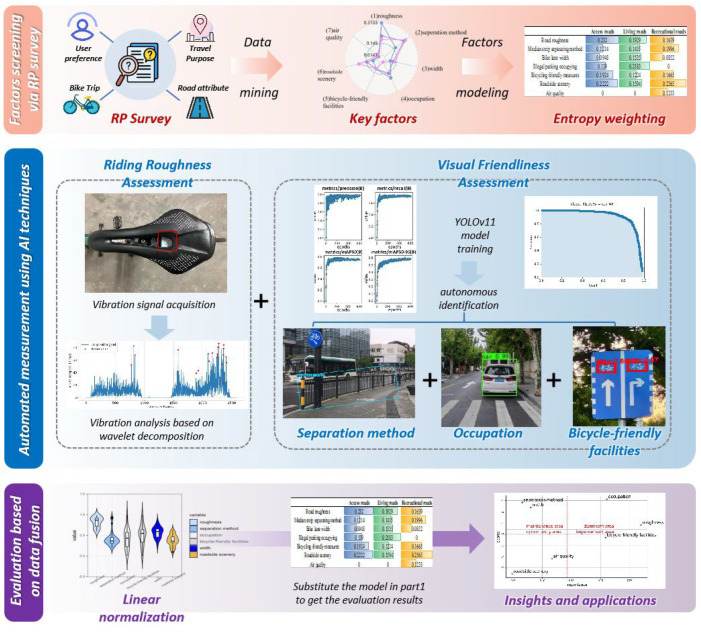
Overarching framework.

Figure 1 illustrates the overall framework.

First, a subjective questionnaire was used to conduct a resident opinion survey to investigate the influence of each element on cycling friendliness. The GBDT significance analysis method [29] was applied to filter factors and retain important elements, including road roughness, median separation method, cycle lane width, illegal parking, cycling-friendly measures, roadside scenery, and air quality. Then, based on the entropy value method, the weights of the elements were determined, and a subjective model was established. Through targeted questionnaire design, the evaluation system accounts for variations in road types and functions.

For objective data acquisition and processing, key indicators of bike lanes are assessed through data collection and analysis methods. This mainly includes using signal processing techniques to evaluate road roughness and image recognition technology to extract target objects for evaluation. The processed data are then converted into impact scores through mathematical modeling.

After normalizing the scores for each component, the scores for each element were substituted into the subjective evaluation model developed in the first step to derive a comprehensive road evaluation that provides a critical foundation for road maintenance, enhancement, and informed cycling decisions by residents. Enhanced service quality and wiser options presented to users are expected to increase cycling usage rates.

### 2.2. Bicycle-Friendly Evaluation Using RP Survey Analysis

#### 2.2.1. Questionnaire Survey and Results Processing

This study uses questionnaires to collect research data. The road samples in this study are derived from the Open Street Map [30]. In the sampling process, the two key elements of road type and geographic location are considered together, and the scientific method of stratified sampling is used for accurate sampling. The selected roads are categorized into three types—access, living, and recreational—by primary usage, and representative sections were selected for designing the questionnaire. The questionnaire is divided into three parts. The first part gathers basic information, such as the frequency and purpose of cycling, helping to identify a representative cyclist sample. The second part provides an overall evaluation of roads based on cyclists’ ratings. In contrast, the third part assesses the level of influence of various factors, where cyclists select and evaluate key factors of bicycle friendliness across multiple dimensions.

Two sampling methods were employed for respondent selection: interception sampling and stratified sampling. Interception sampling, a form of convenience sampling, involves randomly intercepting passers-by on selected roads to conduct surveys. Stratified sampling was used to select residents’ households based on residential neighborhoods. This method followed established stratification criteria, ensuring systematic and representative selection of individual residents. Together, these approaches ensured that the sample was both scientifically rigorous and representative, meeting the study’s diverse needs. Cyclists select from five impact levels: no impact, slight impact, moderate impact, high impact, and very high impact. Respondents used a sliding bar to drag and select values between 0 and 100 for evaluation. A trap question at the end, asking for the administrative district of the evaluated road, screened out cyclists who failed the survey criteria or did not participate attentively. Images illustrated each factor’s impact on cycling friendliness, helping ensure respondent understanding. A total of 2651 questionnaires were distributed, yielding 2537 valid responses.

To improve data credibility, responses failing trap questions were excluded, and a comparative analysis was conducted to ensure logical consistency, eliminating evident errors. Regression analysis was initially conducted to establish a linear equation, transforming data to reflect differences between questionnaire and model-generated scores. Responses with an absolute deviation exceeding 25 were removed, resulting in the final valid questionnaire set. This process also screened inconsistencies between initial and subsequent responses, with reliability testing confirming data suitability for further analysis. Reliability reflects a measure’s ability to produce stable results consistently. Cronbach’s Alpha (α), widely used for assessing internal consistency, is particularly suitable for Likert scales. For exploratory studies, a reliability threshold of 0.60 or higher is recommended, with classifications: excellent (0.90 and above), high (0.70–0.90), moderate (0.50–0.70), and low (below 0.50) [31]. After initial data processing, a total score was calculated through regression analysis, and each questionnaire’s absolute score deviation was measured. Deviations were classified as follows: 5 points (within 5), 4 points (5–10), 3 points (10–15), 2 points (15–20), and 1 point (20–25). Using this approach, reliability across multiple roads was assessed, with partial results shown in Table 1. As shown in Table 1, reliability scores for each road exceeded 0.6, indicating satisfactory usability of the questionnaire.

After verifying the questionnaire’s validity, it was essential to confirm that the data were suitable for factor analysis, a key step in subsequent analyses. Two primary tests—Kaiser–Meyer–Olkin (KMO) and Bartlett’s test of sphericity—are commonly used to evaluate this suitability [32]. The KMO test measures whether variable correlations are adequate for factor analysis, with values closer to 1 indicating stronger inter-variable correlations and, thus, greater suitability for factor analysis. Generally, a KMO value above 0.6 or 0.7 is deemed acceptable for this purpose. Conversely, Bartlett’s test of sphericity checks whether the correlation matrix is an identity matrix, where a *p*-value below 0.05 implies significant correlations among variables, affirming the data’s compatibility with factor analysis.

In this study, both KMO and Bartlett’s tests were conducted before statistical modeling. The KMO value above 0.8 demonstrates strong inter-variable correlation, suggesting high suitability for factor analysis. This study checked the data against the results of the surveys for the different roads by performing Cronbach’s alpha, KMO, and Bartlett’s test of sphericity. Table 1 presents the results of some of the questionnaire data. Questionnaires that did not meet the established criteria were considered unsuitable for modeling; other investigations were conducted before the data met the required criteria. In addition, Bartlett’s test (Bartlett’s test) yielded a significance level of less than 0.05, confirming the existence of significant differences between the measured items, further supporting the factor analysis.

**Figure 2:**
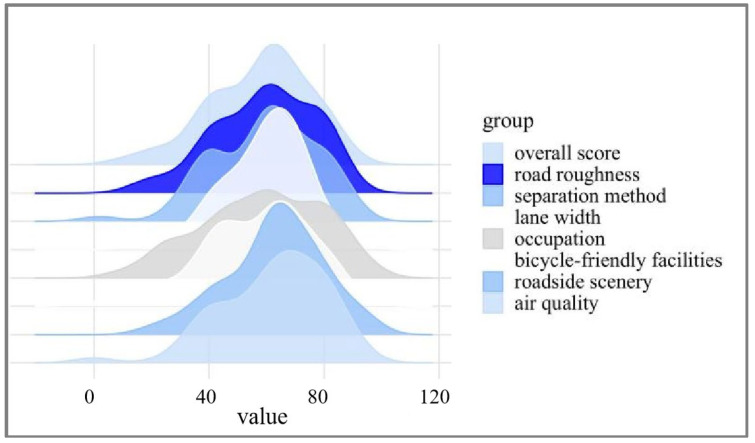
The distribution of each variable.

Beyond these tests, data distribution was also assessed to verify data validity. High deviations or an uneven distribution could indicate reliability issues or data processing errors, warranting potential reevaluation of the study. In this case, the data distribution, illustrated in Figure 2, follows a usual trend, indicating appropriate distribution and affirming the dataset’s reliability for subsequent analyses. As shown in Figure 2, the data distribution pattern follows a usual distribution trend, indicating an appropriate data distribution. This pattern demonstrates that the dataset is suitable for modeling and supports reliable subsequent analyses.

**Figure 3:**
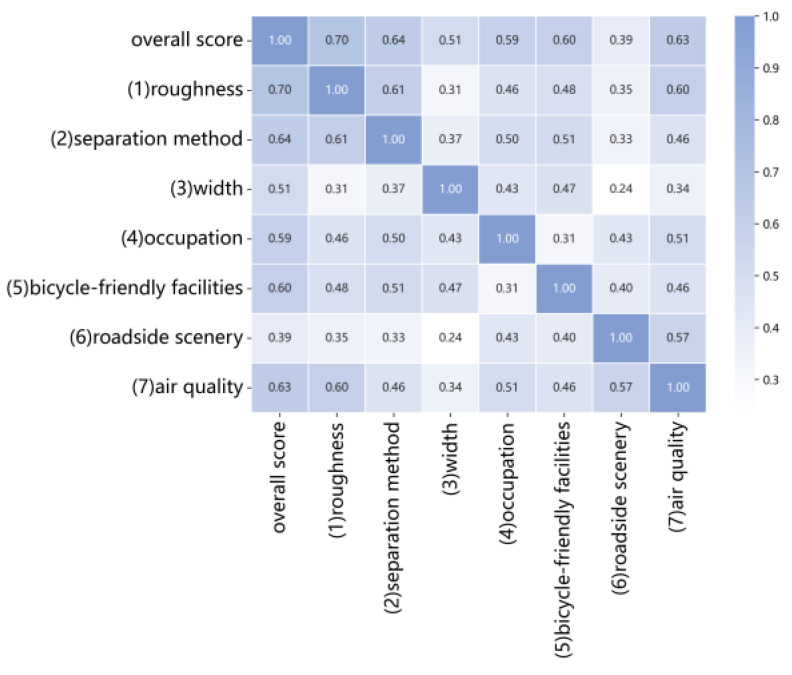
Heat map of the correlation matrix for each variable.

The presence of highly correlated features may lead to redundancy, as similar features might be retained during feature selection. In this study, we utilized questionnaire results to construct the model. Factors were selected based on the data relevance index, and the correlations between variables were analyzed, as shown in Figure 3. The analysis in Figure 3 indicates no strong correlations among the variables, suggesting that all variables are suitable for inclusion in the modeling process.

**Figure 4:**
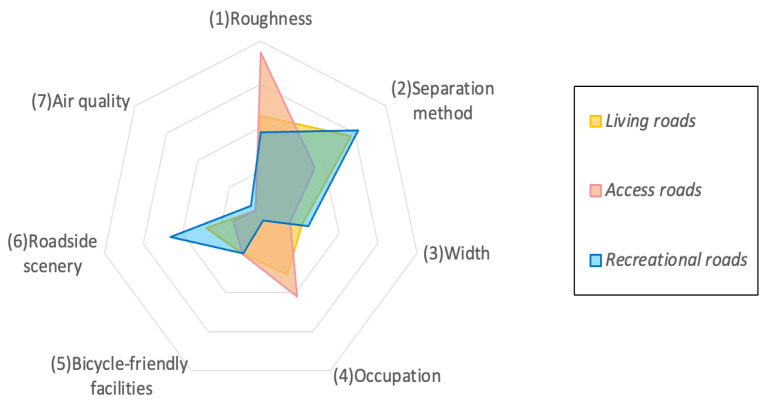
The wind rose diagram of elemental importance of three kinds of roads.

According to the importance of each factor, this study selected the most significant impact indicators as independent variables for building the evaluation system model. GBDT importance analysis is applied to assess feature significance and for the three types of roads. Figure 4 displays the importance of the element in a wind rose diagram.

Based on GBDT analysis results, variables with importance values below 0.2 were considered for removal, as they likely have a limited impact on overall road scoring results. The selected variables for each road type based on GBDT analysis are shown in Table 2.

#### 2.2.2. Quantitative Description Based on Entropy Weighting

The entropy weight method is an objective technique for assigning weights to indicators based on their value variability [33]. Compared to the hierarchical analysis method, which relies on expert judgment to assign indicator weights by comparing their scores, the entropy method is better suited for scenarios with abundant and high-quality data. When sufficient data are available, the entropy method offers broader applicability. In this study, information entropy was used to calculate the entropy weight of each indicator, enabling the establishment of an objective weight set.

Positive indicators are treated according to Equation (1), while negative indicators follow Equation (2), where $x_{ij}$ is the value of the *i*th sample value under the *j*th indicator.

(1) $$x_{ij}=\frac{x_{ij}-\min\left\{x_{ij},\ldots ,x_{nj}\right\}}{\max\left\{x_{ij},\ldots ,x_{nj}\right\}-\min\left\{x_{ij},\ldots ,x_{nj}\right\}},$$

(2) $$x_{ij}=\frac{\max\left\{x_{ij},\ldots ,x_{nj}\right\}-x_{ij}}{\max\left\{x_{ij},\ldots ,x_{nj}\right\}-\min\left\{x_{ij},\ldots ,x_{nj}\right\}},$$

The weight of the *i*th sample value under the *j*th indicator for that indicator (see Equation (3)).

(3) $$p_{ij}=\frac{x_{ij}}{\sum_{i}^{n}x_{ij}},\;i=1,\ldots ,n,j=1,\ldots ,m,$$

The entropy value and the redundancy of the *j*th indicator is calculated as Equations (4) and (5).

(4) $$e_{j}=-k\sum_{i=1}^{n}p_{ij}\ln\left(p_{ij}\right),\;j=1,\ldots ,m,\;\mathrm{where}\;k=\frac{1}{\ln(n)}>0,\;\mathrm{satisfy}\geq 0,$$

(5) $$d_{j}=1-e_{j},$$

Finally, the weight of each indicator is calculated using the redundancy (see Equation (6)).

(6) $$w_{j}=\frac{d_{j}}{\sum_{j=1}^{m}d_{j}}$$

The evaluation model of the three types of roads was obtained, as shown in Table 3.

As illustrated in Table 3, access roads are significantly influenced by the surrounding landscape, as commuters often seek visual enjoyment and psychological relaxation to alleviate daily stress during commutes. Given that the primary purpose of access roads is to connect the starting and endpoints quickly, cyclists are generally less concerned with environmental factors like air quality and instead place more value on the enjoyment of the ride. Living roads, however, face challenges such as roadway occupation by motorized vehicles and illegal parking encroachment, which significantly threaten cyclists’ safety and comfort. Additionally, pavement roughness and road width are critical factors for cycling friendliness on living roads: a smooth road enhances cycling safety. In contrast, a wide road reduces proximity to motorized vehicles or other cyclists, thus minimizing potential conflicts. Recreational roads, in contrast, focus on creating landscapes and segregating motorized from non-motorized lanes. A beautiful landscape offers cyclists a visually pleasant experience, while lane separation ensures a safe and independent cycling environment.

### 2.3. Automated Measurement of Key Bicycle-Friendly Factors

Based on the questionnaire results, the primary factors identified were cycling road smoothness and visual friendliness, which were evaluated, respectively, through signal transformation analysis and image-based object recognition techniques.

#### 2.3.1. Riding Roughness Assessment

Roughness is a significant factor affecting the cycling experience (see Section 2.2.2). Road roughness can result from elements such as speed bumps, surface fractures from aging, or height discrepancies between road sections. Current studies on pavement roughness are mostly based on motor vehicle vibration data, weighting root mean square acceleration and bumps by using maximum acceleration [34,35], and root mean square (RMS) acceleration [36]. The roughness of bike roads also contributes to vibration. Increased roughness in bike lanes leads to a bumpier cycling experience, impacting overall comfort. A vibration-based quantitative method is proposed to assess bike lane roughness, referencing the Highway Performance Assessment Standard (JTG 5210-2018) [37]. Based on the aforementioned Standard, Equation (7) calculates the Road Roughness Index (RRI), which serves as a quantitative method to assess the roughness of a bike lane:

(7) $$RRI=100-a_{0}CBR^{a_{1}},$$

(8) $$s_{1}=CBR=\frac{\beta n}{L}$$

where

$s_{1}$ ——the final value for the factor ‘riding roughness’;

*CBR* ——Cycling bump ratio (%);

$a_{0}$ ——15.00 for asphalt pavement, 10.66 for cement concrete pavement;

$a_{1}$ ——0.412 for asphalt pavement, 0.461 for cement concrete pavement;

β ——Conversion factor (m);

n ——Number of bumps;

L ——Length of the bike lane.

**Figure 5:**
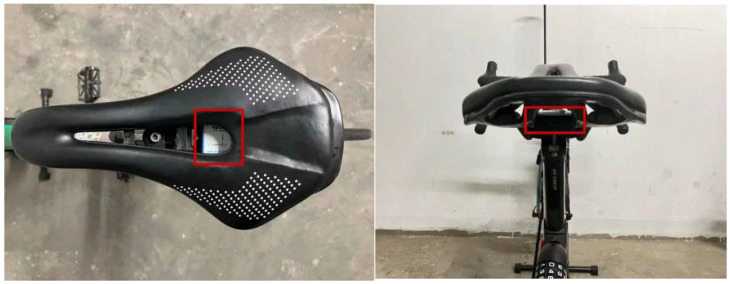
A vibration sensor placed on the bike.

In this formula, bike lane length is directly measurable using a bike computer. A vibration analysis method calculates the number of bumps encountered during cycling. The vibration sensor is mounted on the seat, as shown in the red square frame in Figure 5.

**Figure 6:**
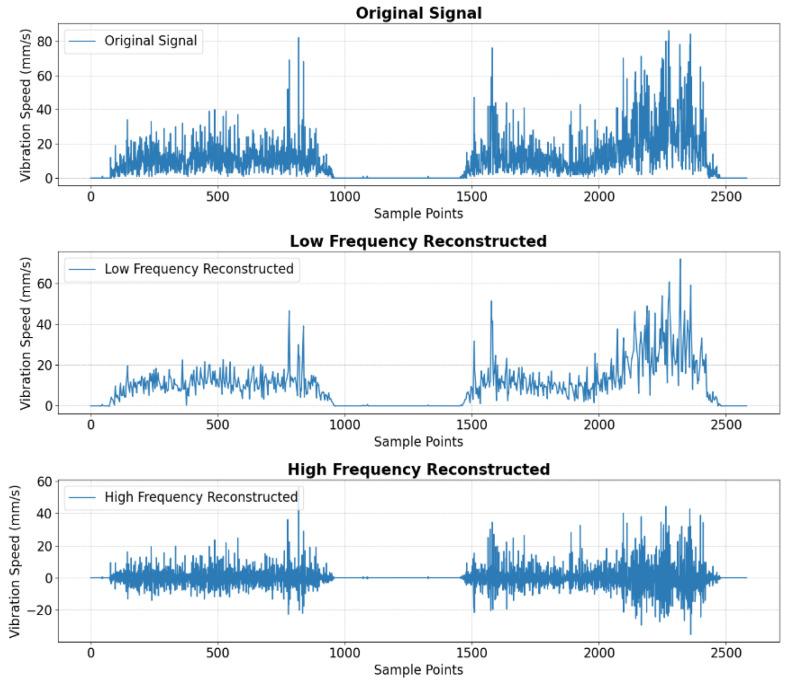
The original signal and the signal reconstructed by Symlet.

This study takes Zhang Wu Road as an example to expose the entire process of vibration signal processing. WTVB01-BT50 vibration sensor with an acceleration accuracy of 0.0005 g/LSB and BSC200 bike computer made by IGPSPORT is used for data acquisition. High-frequency signals primarily represent noise and bumps. Assuming noise at each sampling point follows an independent normal distribution, bumps can be identified by detecting outliers in the high-frequency signal. Extracting road bump information requires filtering and analyzing the high-frequency component of the vibration signal. Wavelet decomposition and reconstruction are widely used in signal processing [38]. This study selects Symlet as the wavelet base for the vibration signal to reconstruct the high-frequency signal, as shown in Figure 6.

After filtering, the high-frequency signal data can be converted into bump counts using the following method. According to the international standard ISO 5349-1:2001 [39], adults experience discomfort at a vibration velocity of 0.5 m/s on vibration levels tolerable to the human body. Therefore, abnormal bumps on cycling routes can be identified if the vibration velocity exceeds this threshold. If vibration velocity is significantly lower than this threshold, abnormal bumps can be detected using the 3σ law of normal distribution. If the signal is not normally distributed, abnormal bumps can be detected using the Z-score method, with calculation formulas shown in Equations (9)–(11).

(9) $$\mu =\frac{1}{n}\sum_{t=t_{0}}^{nt_{0}}S_{t}$$

(10) $$\sigma^{2}=\frac{1}{n}\sum_{t=t_{0}}^{nt_{0}}{\left(S_{t}-\mu \right)}^{2}$$

(11) $$\left|S_{t}-\mu \right|\geq 3\sigma$$

where

t ——time of signal acquisition;

$t_{0}$ ——initial time;

$S_{t}$ ——vibration velocity at time t;

μ ——mean value of the vibration velocity signal;

σ ——standard deviation of the vibrational velocity signal;

n ——number of signal samples.

**Figure 7:**
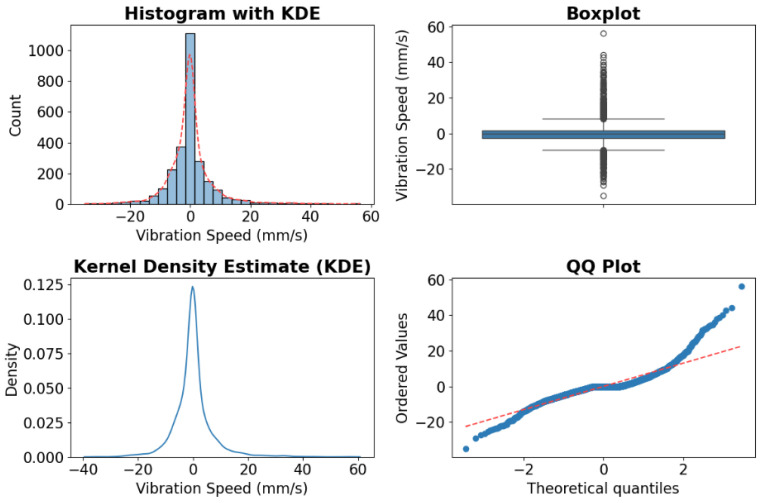
The distribution of the high-frequency signal.

In this example, the high-frequency signal is analyzed, and its distribution is shown in Figure 7. In the figure, the Histogram with KDE (Top Left) displays the distribution of vibration speed data using a histogram. The vertical bars represent the frequency of occurrences for different vibration speeds. The red line overlaid on the histogram represents the Kernel Density Estimate (KDE), which provides a smooth curve that approximates the probability density function of the data. Boxplot (Top Right) provides a graphical representation of the distribution of the vibration speed data through its quartiles. Kernel Density Estimate (Bottom Left) is a standalone version of the KDE shown in the top left plot. QQ Plot (Bottom Right) compares the quantiles of the sample data against the quantiles of the normal distribution. Applying the *p*-value method to test normality in this high-frequency signal indicates that it is not normally distributed. Thus, the Z-score method is used to convert the signal into bump counts.

**Figure 8:**
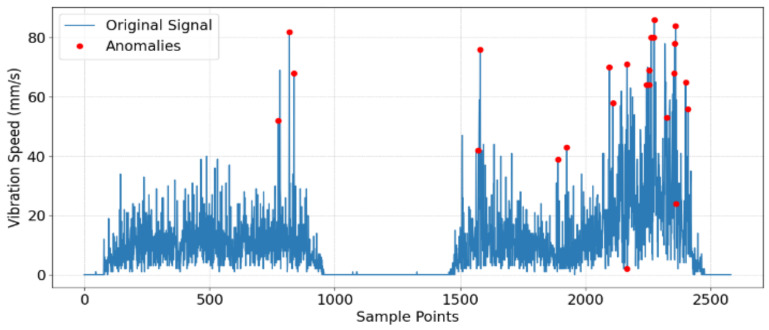
Vibration signal with anomalies marked.

Using a score threshold of 4, 24 outliers in the high-frequency signals are identified, as shown in Figure 8.

Finally, according to the previously mentioned quantitative method, the score (RRI) for this example is calculated as 0.95.

#### 2.3.2. Visual Friendliness Assessment

The visual friendliness of cycling roads refers to the influence of environmental information perceived during cycling. The visual assessment of road visual friendliness primarily involves evaluating the separation methods of motor vehicles and non-motorized vehicles, occupation by parked motor vehicles, and the presence of bicycle-friendly facilities. The critical aspect of evaluating these factors lies in identifying the corresponding elements, a task efficiently performed by the You Only Look Once (YOLO) model.

YOLOv11 [40,41] is employed for the recognition of these elements. It represents an efficient series of object detection algorithms that have achieved significant advancements in recent years. Unlike traditional selective search methods, the YOLO series utilizes a unified neural network architecture that divides an image into grids, predicting bounding boxes and their associated category probabilities [42]. This approach demonstrates excellent real-time performance. Compared with other models or algorithms such as SegNet, Mask R-CNN, and Faster R-CNN, YOLO has a clear advantage in recognition speed [43]. YOLO–OBB is specifically applied to detect the separation method on the target road. Oriented bounding box (OBB) detection extends conventional target detection by incorporating object rotation, enabling the effective identification of inclined objects and enhancing detection accuracy for tilted or oriented targets [44]. This capability makes it more suitable for such tasks compared to axis-aligned bounding boxes. Relevant studies have adopted a two-stage approach to optimize the model, highlighting its significant potential for real-time, large-scale vehicle recognition [45]. This optimized method has proven effective in identifying vehicles from image frames extracted from video data.

In this study, three YOLO models were independently trained to identify different separation methods, occupied vehicles, and bicycle-friendly signs. Due to the absence of existing models for these detection components, training was conducted on thousands of self-categorized and labeled images corresponding to the relevant elements.

**Figure 9:**
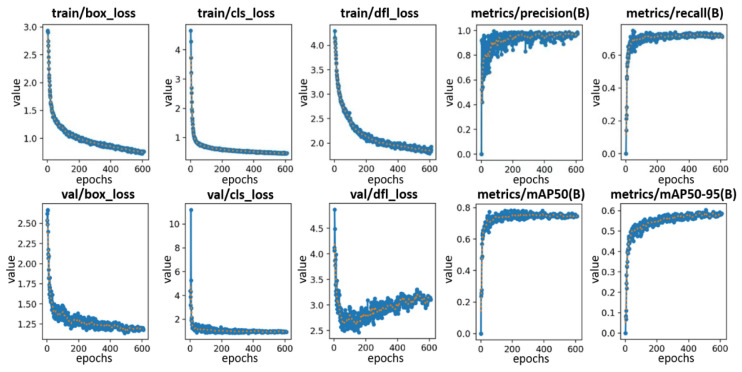
Training results of YOLOv11-OBB.

Field survey imagery from Shanghai was captured and split into an 80% training subset and a 20% validation subset. The training set contained precisely annotated images. For the separation method recognition model, four primary classification scenarios (Green Belt, Guardrail, Line and No separation) were identified through a comprehensive analysis of questionnaire responses and a systematic review of the existing literature. Approximately 3000 images per separation category were annotated, resulting in a cumulative total of 9156 annotated images across all categories (excluding the ‘No separation’ category, which does not require recognition). The occupation recognition model employed the VOC2007 benchmark dataset for vehicle class recognition training, while the friendly facilities recognition model was trained on a dataset containing approximately 15,000 annotated images. Model training was conducted on a computational platform equipped with an NVIDIA GeForce RTX 4060 Laptop GPU running Ubuntu 22.04. The software environment was configured with Python 3.12.7, PyTorch 2.5.0, and CUDA 11.5. The training process utilized an input resolution of 640 × 640 pixels, a batch size of 16, and was conducted over 400 epochs. The Stochastic Gradient Descent (SGD) optimizer was employed for parameter optimization to facilitate efficient convergence. This configuration was selected to balance computational efficiency and model performance. As shown in Figure 9, the separation method recognition model exhibited progressive learning and refinement throughout the training over time. The results demonstrate competitive performance, with reduced loss values and enhanced evaluation metrics.

**Figure 10:**
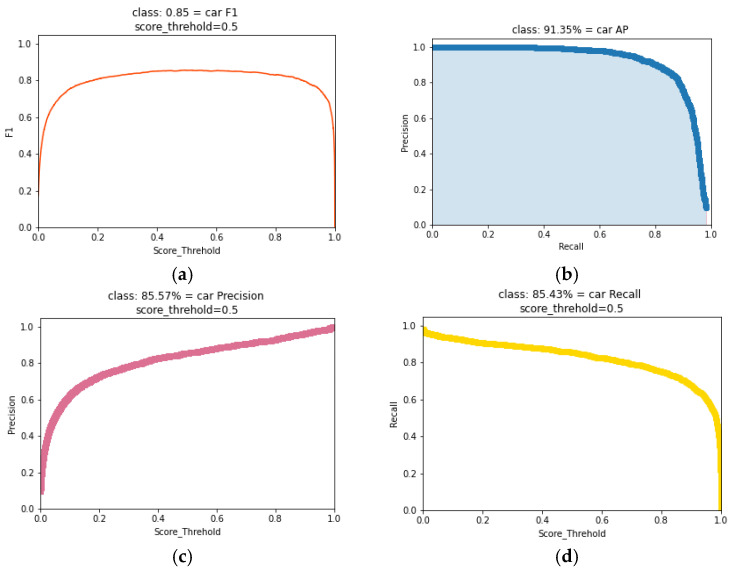
(**a**) F1 value for the ‘car’ class; (**b**) precision–recall curve of ‘car’ class; (**c**) precision–score_threhold curve; (**d**) recall–score_threhold curve.

Techniques like batch normalization and dropout are used to improve classification performance [46,47]. The model is rigorously trained on VOC2007, with results displayed in Figure 10. Figure 10a illustrates the F1 value for the ‘car’ class across varying score thresholds, demonstrating that the F1 score stays consistently above 0.8 when the threshold is set to 0.5, indicating a robust balance between precision and recall. The precision–recall curve in Figure 10b shows an Average Precision (AP) of 91.35% for the “car” class, highlighting the trade-off between precision and recall as recall increases, leading to additional false positives. Figure 10c shows that the model sustains high precision even as the threshold increases. The Recall–Score Threshold curve in Figure 10d complements the precision analysis by demonstrating the model’s capability to detect all instances as the threshold varies. A high recall rate, especially at higher thresholds, indicates that the model effectively captures all instances without omission.

To further enhance recognition accuracy, various image preprocessing techniques, such as Contrast Constrained Adaptive Histogram Equalization (CLAHE) [48] and Spatial Transformer Networks (STNs) [49], are applied to enhance image features and improve the model’s robustness. The accuracy of the various methods employed is presented in Table 4. Ultimately, the best current Single CNN with 3 STNs method was chosen.

Using the trained model, specific elements are identified and utilized to assess the road’s visual friendliness. For the assessment of separation methods, occupations, and friendly facilities, distinct mathematical models are constructed to transform identification results into evaluation scores for each factor.

Regarding the separation method assessment, the YOLO–OBB model is employed to identify different kinds of segmentation methods for evaluation. Motor vehicle and non-motorized vehicle separation facilities are physical barriers that separate motor vehicle and non-motorized vehicle lanes. Their primary function is to prevent motor vehicles from intruding into non-motorized lanes, providing a safer and more comfortable cycling environment. As shown in Section 2.2.2, improved separation facilities significantly enhance bike lane user satisfaction. This study proposes a quantitative methodology to categorize different separation methods. Table 5 categorizes separation methods into four classes to determine their effectiveness in ensuring a safer cycling experience. The higher the separation facility’s grade, the more effective it is at isolating cyclists from the roadway, with a correspondingly higher score.

For multiple separation methods on the same road, a segmented weighted average technique is used to score the separation facilities across the entire road, as shown in Equation (12), where $x_{i}$ represents the average assessed value of separation for this lane, $L_{k}$ is the length of the *k*-th separation method, and k is the score for the *k*-th method, where the score ranges from 0 to 3, as shown in Table 5. This equation assigns weights to the scores of the separation methods based on the length of the corresponding road sections, considering the scenario where a single road section may employ different separation methods. To assess the length of the *k*-th separation method, a video of the entire road section is sampled continuously at a fixed frequency. The number of images represents the length of the *k*-th separation method. Finally, the results are transformed into percentages as in Equation (13), where $x_{min}$ is 0 and $x_{max}$ is 3. $s_{2}$ represents the final value for the factor ‘motor vehicle and non-motorized vehicle separation method’.

(12) $$x_{i}=\frac{\sum_{k=0}^{3}L_{k}\times k}{\sum_{k=0}^{3}L_{k}}$$

(13) $$s_{2}=\frac{x_{i}-x_{\min}}{x_{\max}-x_{\min}}$$

Regarding the occupation assessment, the YOLO model is employed to detect and quantify the number of vehicles occupying bike lanes for evaluation. Vehicle parking occupation on bike lanes refers to motor vehicles that violate traffic regulations by illegally occupying or entering the bike lane space. Such illegal occupation disrupts the continuity of bike lanes, and survey results indicate that a higher incidence of motor vehicles occupying bike lanes reduces the road’s cycle-friendliness (see Section 2.2.2). Consequently, the number of vehicles is utilized as a metric to establish the formula (see Equation (14)), where n is the number of motor vehicles illegally parked in bike lanes, L is the road length, and $x_{i}$ is the occupation level. The evaluation score is calculated by the number of motor vehicles illegally parked in bike lanes divided by the road length, with units of vehicles/km. The more vehicles occupying the cycling lane, the lower the road section’s cycle-friendliness. Since this encroachment is a negative indicator, it is adjusted using Equation (15). In this formula, $s_{3}$ represents the final score for the factor ‘bike roads occupied by unauthorized vehicles’. $x_{max}$ is the maximum value in this evaluation. Considering that an average vehicle is approximately 4.5 m long, $x_{max}$ is set to 1000/4.5 = 220 vehicles/km, indicating that the road is fully occupied by unauthorized vehicles. The minimum evaluation value, $x_{min}$, is assumed to be 0, indicating the road is entirely unoccupied.

(14) $$x_{i}=n/L$$

(15) $$s_{3}=\frac{x_{\max}-x_{i}}{x_{\max}-x_{\min}}$$

**Figure 11:**
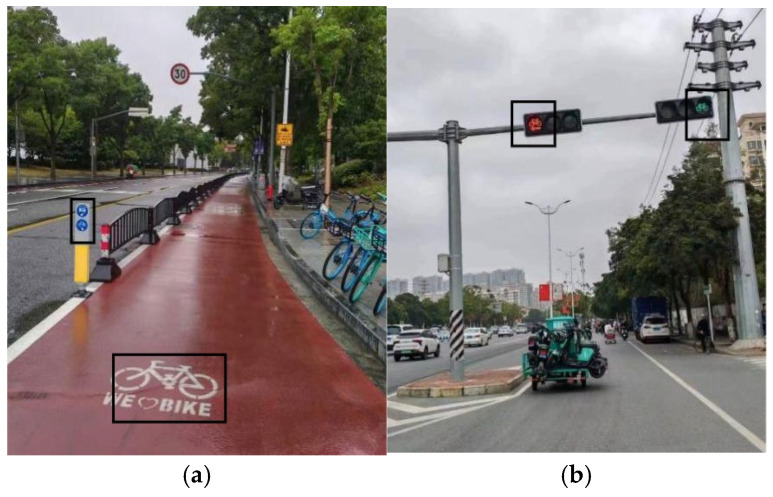
(**a**) Static cycle-friendly sign; (**b**) dynamic cycle-friendly signs.

Regarding the friendly facilities assessment, the YOLO model is employed to detect and quantify the number of cycle-friendly signs for evaluation. Cycle-friendly facilities refer to supportive signs and markings that offer guidance and support for cyclists. These facilities are categorized into two types: static, including bicycle markings, signage, and guidelines, and dynamic, such as bicycle signals and left-turn phases. Figure 11 illustrates an example of cycle-friendly facilities.

There is a direct correlation between cycle-friendly facility density and cycling friendliness. An increased presence of friendly signs not only effectively guides cyclists on their routes but also enhances their confidence in road safety, encouraging more people to choose cycling. When bike lanes are clearly and adequately marked with cycle-friendly signs, it helps enhance cyclists’ sense of safety and contributes to a better overall experience. In this study, this metric is evaluated based on the density of friendliness markers. Friendly facility coverage density is expressed by Equation (16), where n represents the number of signs and L is the length of the roadway. $s_{4}$ represents the final score for the factor ‘bicycle-friendly facilities’. This indicator assesses the score for bicycle-friendly facilities using the number of signs per unit road.

(16) $$s_{4}=n/L$$

**Figure 12:**
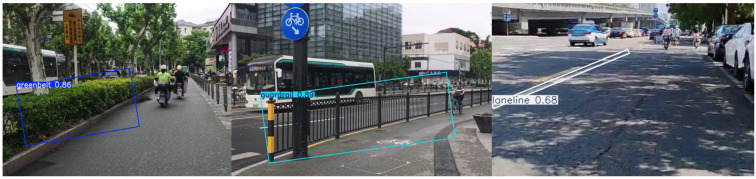
Detection results of the separation method.

**Figure 13:**
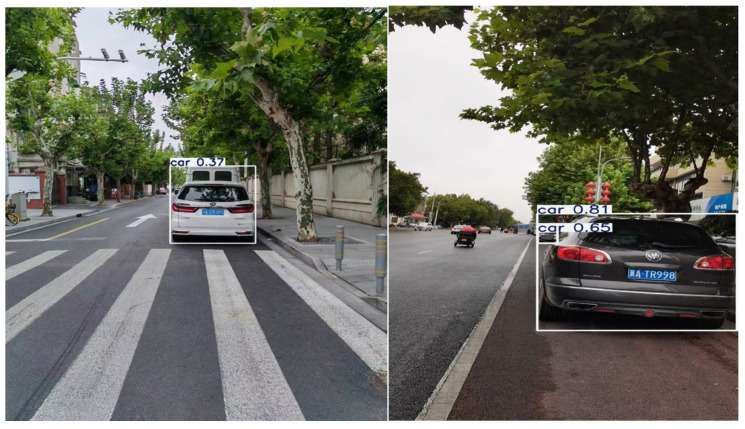
Detection results of lane occupation.

**Figure 14:**
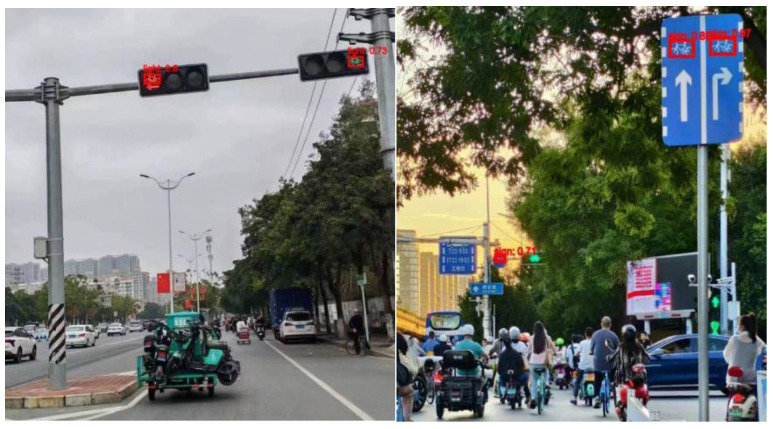
Detection result of cycle-friendliness facilities.

The trained model performs well in recognizing elements related to visual friendliness. The output image displays multiple bounding boxes with identified names and confidence values, as shown in Figure 12, Figure 13 and Figure 14, accurately identifying the locations and classes of the objects. This accurate detection makes it possible to evaluate relevant factors of bike lanes.

In addition to the above points, this study also considers the impact of green space along roadways on cycling visual friendliness. As residents’ expectations for travel experiences grow, green space along roadways is increasingly vital in enhancing cyclist comfort and well-being, particularly for riverfront leisure-oriented bike lanes [54,55]. This study adopts the road green coverage ratio as a primary metric for evaluating the cycling landscape, assuming that a higher percentage of green areas within the field of view will improve ride friendliness. The green area coverage rate is defined in Equation (17), where $S_{green}$ represents the area of green belt pixels and $S_{total}$ is the total image area. $s_{5}$ represents the final score for the factor ‘roadside scenery’. This study utilizes the CV2 library in the OpenCV framework for image processing. By identifying pixels within a defined RGB color range, the ‘inRange’ function creates a binary mask, isolating green regions within the image [56]. This approach segments vegetated areas, such as green belts or plant-covered surfaces, using color thresholds, enabling precise assessment of green coverage.

(17) $$s_{5}=S_{green}/S_{total}$$

The evaluation of road width adheres to China’s Urban Road Engineering Design Code, which specifies dimensional standards based on road classification and functional type. In this study, road width data (denoted as $s_{6}$) were directly extracted from the Baidu Maps Open Platform [57]. $s_{6}$ represents the final score for the factor ‘road width’.

### 2.4. Bicycle-Friendly Evaluation Based on Data Fusion

Utilizing the aforementioned evaluation framework, quantitative scores for each road attribute were systematically derived. The variables s1 to s6 represent the evaluated scores for roughness, separation, occupation, friendly facilities, scenery, and width, respectively. However, significant scale discrepancies among these parameters impede the direct comparability of raw scores. As a result, the direct incorporation of unprocessed scores into the evaluation model is technically infeasible. To address this issue, a normalization procedure was implemented to standardize all metrics, followed by weighted aggregation based on predefined coefficients derived from prior analyses.

**Figure 15:**
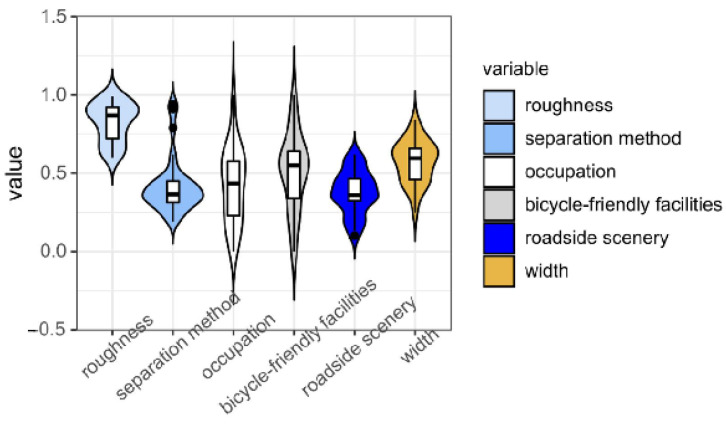
Distribution of values in relative indicators.

In this study, linear normalization was adopted. Distribution functions were statistically derived from parameter value distributions to establish categorical thresholds. A representative sample of 107 roads representing diverse road conditions in Shanghai, China, was randomly selected for evaluation using the methodology outlined in Section 2.3. Figure 15 presents the empirical distributions of evaluation metrics. The random sampling strategy resulted in evaluation outcomes exhibiting approximately normal distributions, demonstrating the inherent heterogeneity in urban road networks. While the scores for most elements approximately conform to a normal distribution, the data ranges vary significantly due to differences in evaluation methods. For example, roughness scores are systematically higher than those for the road environment, reflecting the distinct measurement scales and criteria applied to each factor.

To mitigate the impact of such methodological disparities, the 99% confidence interval of the normal distribution was adopted as the normalization threshold, which serves as the values of $S_{ihigh}$ and $S_{ilow}$ (as shown in Table 6). This statistical approach ensures the exclusion of extreme values while preserving the central tendency of the data, thereby enhancing comparability across heterogeneous indicators.

The six elements for each road are identified using the method described in Section 2.3, and the relative score $s_{i}$ is calculated (see Equations (8), (13) and (15)–(17)). These scores were subsequently normalized to derive $S_{ifinal}$ (see Equation (18)). $S_{ifinal}$ denotes the final evaluation score for each factor after data fusion.

(18) $$S_{ifinal}=\frac{s_{i}-S_{ilow}}{S_{ihigh}-S_{ilow}}$$

Finally, based on these calculations, roads are classified as access, living, or recreational, with $S_{total}$ obtained by substituting $S_{ifinal}$ into the model formula (see Equation (19)). For the three road types, the weights of the individual elements $w_{i}\;$ are determined in Section 2.2.2 (given in Table 3 above).

(19) $$S_{total}=\sum_{i=1}^{6}w_{i}*S_{ifinal}$$

The final evaluation results provide valuable guidance for cyclists in selecting optimal routes. Additionally, the results encompass both scores and their relative importance, which together provide valuable guidance for future improvements.

## 3. Case Study

### 3.1. Data Access and Process

As a mega-city in China, Shanghai has made great efforts to develop green transport in recent years, building many kinds of bike lanes. In this study, a total of ten roads of various types were selected to apply the method from Shanghai, China, based on real-time map database information. Field riding tests were conducted, and video data of the test roads were obtained using a motion camera mounted on a bike with a fixed position and horizontal angle. Vibration data were collected using WTVB01-BT50 vibration sensors and a BSC200 yardstick. Weather data were sourced from 2023 statistics from the Shanghai meteorological station.

To facilitate recognition analysis, video data were converted into images by extracting frames at a set frequency. The OpenCV library was used to load the video file and extract frames. This method loops through each frame, saving selected frames at a defined interval for analysis. Data preprocessing is essential to enhance the accuracy of subsequent analysis and includes Gaussian filtering, median filtering, mask generation, and morphological processing.

Vibration signals require preprocessing before analysis, including the following steps:

Step 1: Missing value treatment—missing values were filled using data from the previous time point.

Step 2: Outlier handling—a fixed threshold is applied for detection, and outliers are replaced by linear interpolation (see Equation (20)).

(20) $$V_{t}=V_{t-t_{0}}+\frac{V_{t+t_{0}}-V_{t-t_{0}}}{2t_{0}}t_{0}$$

where

t——time at which the abnormal signal is detected;

$t_{0}$——time interval during signal acquisition;

$V_{t}$——vibration velocity at time.

### 3.2. Indicator Scoring and Bicycle-Friendly Assessing

**Figure 16:**
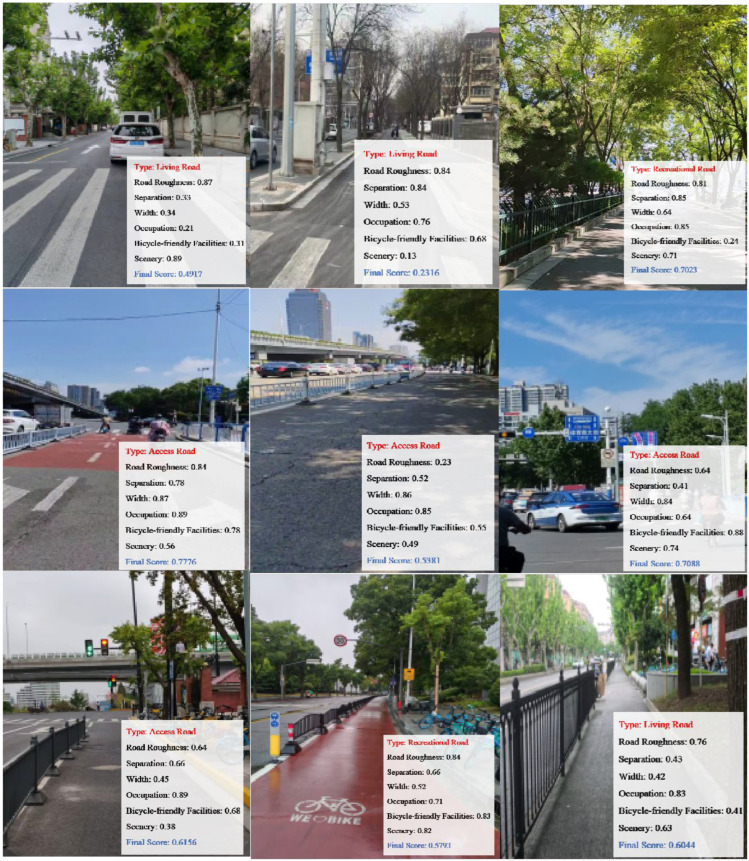
Field tests and results analysis.

Based on the field data collected, the bicycle-friendly factors were evaluated and scored separately according to the road type. As shown in Section 2.3, the vibration signals were utilized to calculate the road roughness score ($s_{1}$), while the image video data were employed to derive the visual friendliness scores ($s_{2}$–$s_{5}$). Moreover, the road width ($s_{6}$) score was obtained from the query values. The final score was computed by integrating the factor weights with their respective scores (see Equation (19)). Figure 16 presents the final scores for the nine target road sections.

### 3.3. Assessing Results Testing and Application

**Figure 17:**
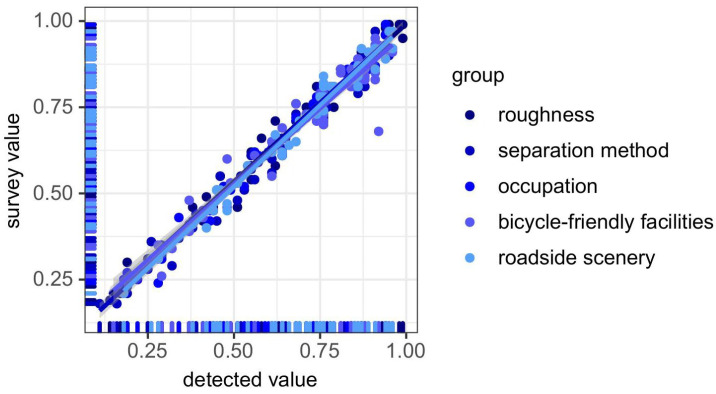
Regression results between detected and surveyed values.

Sixty unique participants were recruited to rate each road’s perception score for each of the factors which have been mentioned above on a scale from 0 to 1. Survey results show a strong positive correlation between test and survey values, with a regression slope close to 1. The goodness of fit of each element ($R^{2}$) is shown in Table 7. No visible misalignment is observed in the data distribution ($R^{2}$ > 0.81) (see Figure 17), supporting the validity of the testing methods proposed in this study.

**Figure 18:**
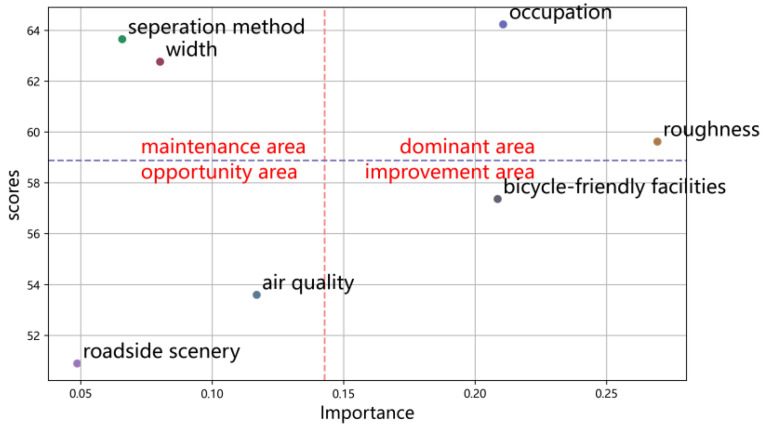
The application analysis of the Zhangwu Road evaluation results.

For specific roads, Changwu Road was selected as a representative case study to validate the framework’s applicability. Following the evaluation process, the importance rankings and quantitative scores of each element were spatially mapped, as shown in Figure 18. In contrast to the weights calculated using the entropy method, the importance values obtained from the questionnaire survey more accurately reflect the subjective significance of road condition elements. Importance denotes the degree to which an element impacts cycling friendliness, as derived from the questionnaire survey (see Section 2.2.1). The scores represent the evaluation outcomes of each element, obtained through automated measurements of objective data (including vibration and image data). Given that the application process emphasizes the relative magnitudes of importance and scores, the mean value serves as a threshold to partition regions. The Dominant zone represents elements that are both crucial and highly rated, thus, should be maintained as strengths; the Improvement zone represents elements that are of high importance but have low scores, indicating areas that should be prioritized for improvement; and the Opportunity zone reflects elements with low scores and low importance, suggesting potential areas for future enhancement.

Based on the results in Figure 18, road roughness and vehicle occupancy conditions are favorable and can be emphasized as strengths, while measures for cycle-friendly facilities are in urgent need of improvement. Enhancements to cycling scenery should also be prioritized within budgetary constraints. Improving these key indicators enhances the overall cycling friendliness of bike lanes, thereby encouraging residents to adopt non-motorized transportation, increasing bicycle usage frequency, and contributing to the advancement of more sustainable transportation systems.

## 4. Conclusions

This study proposes a novel multi-data fusion framework that addresses the limitations of existing approaches, which often rely exclusively on either subjective or objective methods and fail to account for road variability or evaluation comprehensiveness. By integrating subjective perceptions with objective metrics, the framework enables a holistic assessment of cycling infrastructure. It incorporates user-centered questionnaires to align with residents’ expectations, creating a context-aware evaluation system. The framework categorizes road types for tailored modeling, defines specific evaluation indices, and employs machine learning for automated detection. Furthermore, the integration of vibration signal processing and computer vision techniques supports precise and scalable assessments of large-scale road networks.

The proposed framework exhibits strong performance, with classification accuracy ranging from 85% to 91% at a 0.5 threshold, along with high predictive accuracy. The method’s validity is further supported by employing the rider’s first-view perspective for data detection, ensuring high accuracy in data processing, and establishing high consistency ($R^{2}$ > 0.81) between objective and subjective evaluations as assessed by 60 subjects who scored road conditions after actual rides. The framework’s capability to process large datasets efficiently through advanced computational techniques guarantees scalability and applicability in diverse urban settings.

This study offers actionable insights for upgrading and redesigning bike lanes, making a significant contribution to advancing urban planning practices. It provides a robust tool to address the diverse needs of road users, fostering the development of safer, more efficient, and sustainable bike lanes. The findings can directly guide cyclists in selecting optimal routes and support enhancements to road cycling infrastructure, ultimately improving the cycling experience and encouraging its adoption as a mode of travel. Unlike reward-based incentives [58], this methodology promotes cycling by addressing fundamental aspects of road service quality, demonstrating substantial practical value. Additionally, it supports ongoing urban mobility initiatives and establishes a foundation for future innovation in AI-driven urban evaluation systems.

However, the method’s applicability is limited to cities with similar characteristics. Extending it to cities with diverse road systems or scales requires collecting user evaluation data through surveys, necessitating further refinement. Additionally, data detection issues may arise from low-resolution cameras or incorrectly mounted vibration sensors. However, given the high accuracy achieved in image recognition in this study, it is assumed that the error can be minimized through repeated testing and the integration of crowdsourced data.

Future research should emphasize the utilization of crowdsourced data by deploying integrated sensing devices on shared bicycles and incorporating them into the platform’s backend system. Crowdsourcing enables diverse users to perform repeated assessments of the same road segments utilizing heterogeneous equipment, thereby enhancing the statistical reliability of evaluations and positioning this methodology as a scalable approach for large-scale assessments. However, several challenges persist, including bicycle sensitivity to small cracks, the influence of rider weight, temperature-dependent tire performance, and the complexity of road surfaces. These factors complicate vibration data analyses, necessitating further research to ensure reliable road health monitoring.

## Figures and Tables

**Table 1:** Partial calculated results of KMO and Bartlett’s test.

ROAD	Α	KMO	BARTLETT’S TESTS
CaO’an Highway	0.65	0.8612	1.8121 × 10^−3^
Changwu Road	0.73	0.8609	1.5022 × 10^−2^
Guofu Road	0.89	0.8125	1.8560 × 10^−2^
NANJING ROAD	0.67	0.8569	1.8233 × 10^−4^
Linhong Road	0.72	0.7988	1.7524 × 10^−2^
Wujiang Road	0.85	0.8549	1.6526 × 10^−3^
Shengxin Road	0.69	0.8851	1.8413 × 10^−3^
Gaotai Road	0.78	0.7875	1.8426 × 10^−2^
Hezuo Road	0.85	0.8578	1.5492 × 10^−2^
Yunping Road	0.76	0.8916	1.5155 × 10^−4^

**Table 2:** Description of variables.

The Type of Model	Variables
Access roads	Road roughness
	Motor vehicle and non-motorized vehicle separation method
	Bike lane width
	Occupation by parked motor vehicles
	Bicycle-friendly facilities
	Roadside scenery
Living roads	Road roughness
	Motor vehicle and non-motorized vehicle separation method
	Bike lane width
	Occupation by parked motor vehicles
	Bicycle-friendly facilities
	Roadside scenery
Recreational roads	Road roughness
	Motor vehicle and non-motorized vehicle separation method
	Bike lane width
	Bicycle-friendly facilities
	Roadside scenery
	Air quality

**Table 3:** Results of weight calculation by entropy method.

	ACCESS ROADS	LIVING ROADS	RECREATIONAL ROADS
ROAD ROUGHNESS	0.232	0.1929	0.1639
MEDIAN STRIP SEPARATING METHOD	0.1214	0.1435	0.1996
BIKE LANE WIDTH	0.0948	0.1535	0.0852
ILLEGAL PARKING OCCUPYING	0.139	0.2183	0
BICYCLING-FRIENDLY MEASURES	0.1924	0.1224	0.1665
ROADSIDE SCENERY	0.2222	0.1594	0.2565
AIR QUALITY	0	0	0.1233

**Table 4:** Recognition rate accuracy of various methods on GTSRB.

Method	Accuracy (%)
Single CNN with 3 STNs [49]	99.71
HLSGD (20 CNNs ensemble) [50]	99.65
GDBM [51]	99.34
Human performance (best) [52]	99.22
OneCNN [53]	99.11 + 0.10

**Table 5:** Scoring criteria for bike lane separation facilities.

Grade	Good	Fair	Poor	None
Class	Green Belt	Guardrail	Line	No separation
Score	3	2	1	0

**Table 6:** Upper and lower limits for normalization of variables.

Variables	Roughness	Separation	Occupation	Friendly Facilities	Scenery	Width
i	1	2	3	4	5	6
$S_{ihigh}$	0.88	0.51	0.53	0.63	0.47	0.64
$S_{ilow}$	0.76	0.33	0.29	0.39	0.31	0.51

**Table 7:** Evaluation correctness test.

Variables	Roughness	Separation	Occupation	Friendly Facilities	Scenery
$R^{2}$	0.84	0.83	0.89	0.88	0.81

## Data Availability

The data presented in this study are available on request from the corresponding author due to privacy.
